# Antibiotics in Surface Sediments from the Anning River in Sichuan Province, China: Occurrence, Distribution, and Risk Assessment

**DOI:** 10.3390/toxics12060411

**Published:** 2024-06-04

**Authors:** Junlie Zhou, Jianglin Kang, Chunyan Lin, Qi Xu, Wanrong Yang, Ke Fan, Jinrong Li

**Affiliations:** 1 School of Environment & Resource, Xichang University, Xichang 615000, China; 2 School of Sciences, Xichang University, Xichang 615000, China

**Keywords:** antibiotics, sediments, the Anning River, ecological risk assessment

## Abstract

The occurrence, distribution, and ecological risk assessment of 36 antibiotics from five groups, including macrolides (MLs), fluoroquinolones (FQs), tetracyclines (TCs), amphenicols (APs), and sulfonamides (SAs), were investigated for the first time in the Anning River, Sichuan Province, China. The results show that antibiotics were widely present in the sediments of the Anning River, with a total of 22 antibiotics detected. FQs were among the most abundant antibiotics, followed by TCs, MLs, APs, and SAs. The total concentrations of antibiotics in surface sediments varied from 0.05 to 53.35 ng/g, with an average of 8.09 ng/g. Among these groups, MLs, FQs, and TCs emerged as the predominant classes of antibiotics. The midstream sediments showed the highest residual levels of antibiotics, with lower levels observed in the downstream and upstream sediments. Anthropogenic activities, such as human clinical practices and animal breeding, might be sources of antibiotics released into the river. An ecological risk assessment revealed that trimethoprim from the SA group exhibited high risks, and MLs showed medium risks in the Anning River, whereas most antibiotics presented minimal to low risks. This study provides valuable information on antibiotic pollution in the upstream region of the Yangtze River, and future management measures are needed for the Anning River.

## 1. Introduction

Antibiotics have been widely employed in both veterinary and human medicine due to their exceptional ability to treat infectious diseases, as well as their role in promoting growth in aquatic animals, livestock, and plants [1,2,3]. During its rapid growth, China has emerged as one of the leading global producers and users of antibiotics, with a total antibiotic consumption of 162,000 tons in 2013 [4,5,6], which is approximately 150 times greater than that of the United Kingdom [7]. Most antibiotics cannot be completely metabolized by organisms, and up to 85% of these antibiotics or their metabolites can enter surface water and sediment via the excretion of urine and feces, household waste, livestock, and agricultural wastewater [8,9]. Additionally, the widespread usage of antibiotics has negative effects on various organisms and ecosystems. It is possible that they can cause phytoplankton toxicity, inhibit the growth of microorganisms, and alter the structure of microbial communities [10]. Furthermore, exposure to antibiotics may promote the development of antibiotic-resistant bacteria and genes [11,12], raising serious concerns regarding animal and human health through food consumption [13].

Owing to the mismanagement of antibiotics, they are widely distributed in surface water [14,15,16]. Upon entering the water, antibiotics have the potential to be harmful to aquatic organisms at even minimal concentrations (ng/L or μg/L) [17,18,19,20]. Previous studies have suggested that sediments serve as significant repositories for antibiotics [21,22,23] and may also act as potential secondary reservoirs of these contaminants [14,15]. It is natural for many chemicals to accumulate in sediments, which form habitats for plants and animals [24]. Releasing antibiotics into water can potentially act as a source under hydraulic conditions and aquatic physicochemical properties, including pH, organic carbon content, and the presence of metal ions [16,25,26]. Their residual levels in sediments can provide information about long-term pollution levels in an area [27]. Hence, it is essential to analyze the pollution attributes of antibiotics in sediments and assess their potential effects on aquatic ecosystems. In recent years, numerous studies have been conducted on the occurrence, distribution, and risk assessment of antibiotics in sediments in China, such as the Yongjiang River [28], Pearl River, Yellow River, Hai River, Liao River [29], Hanjiang River [14], and Yangtze River [27]. In addition, Sichuan Province is a key region for economic development and environmental conservation upstream of the Yangtze River. Among the 10 typical rivers in Sichuan Province, the levels of antibiotics in the Minjiang River, Jialing River, and Qujiang River exhibited higher concentrations and posed more significant ecological risks compared to other regions [30]. However, relevant studies on the Anning River remain limited.

The Anning River flows through four counties and finally runs into the Yalong River Basin, one of the seven major river basins in China. It covers an area of 11,150 km^2^ and has a river length of ~350 km. The topography is marked by higher latitudes in the northwest and lower latitudes in the southeast. The Anning Valley Great Plains rank as the second largest plains in Sichuan Province [31]. Within the basin drainage area, the river serves not only as the primary source of drinking water for the towns along the river but also as an important water source for agricultural irrigation. Therefore, it is necessary to conduct a systematic and comprehensive study of the occurrence, distribution, and risk assessment of antibiotics in the surface sediments of the Anning River. Moreover, the stability of analytes is a critical point in chemical analysis, especially in the field of trace levels of antibiotics residue analysis [32]. This study has implemented rigorous sample collection and pretreatment methods, as well as strict quality control and assurance measures for antibiotic analysis in sediments, in order to ensure the stability of analysis results. Our study offers a comprehensive insight into the presence of antibiotics in the main river of Sichuan Province and offers theoretical support for safeguarding the river’s water quality.

## 2. Materials and Methods

### 2.1. Sample Collection

**Figure 1:**
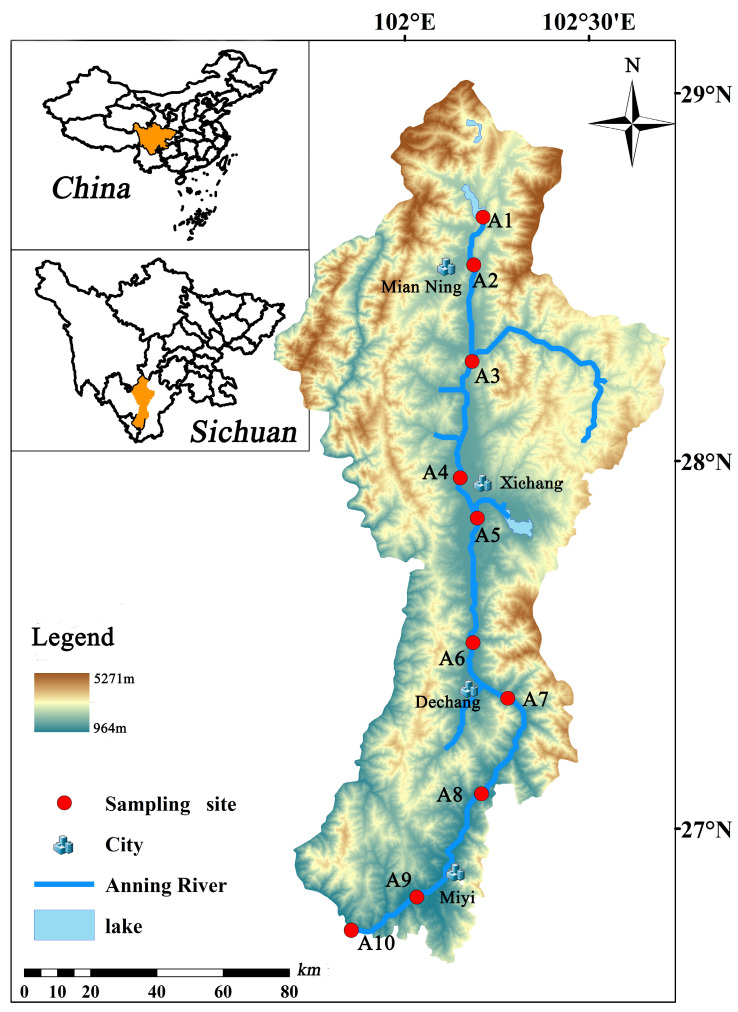
Location of sampling sites in the Anning River.

During the month of April in 2023, a total of ten surface sediment samples were collected from ten different locations along the Anning River, in conjunction with field research and an examination of pertinent information (Figure 1 and Table 1). Each sample was collected from the top 5 cm of the river sediments using a core sampler and was partitioned into two subsamples. The surface sediments were collected with a stainless-steel shovel that had been cleaned thoroughly. After sampling, the samples were wrapped in aluminum foil that had been rinsed with solvent, placed in a cooler, and transported to the laboratory within 24 h. They were then stored at −20 °C in darkness until they could be further processed. Subsequently, the samples underwent freeze-drying, milling, and sieving (0.25 mm). The treated sediment samples were then stored in opaque glass containers.

### 2.2. Chemicals and Reagents

This study focused on 36 antibiotics from five different categories: macrolides (MLs), fluoroquinolones (FQs), tetracyclines (TCs), amphenicols (APs), and sulfonamides (SAs). The MLs included Azithromycin (AZM), Erythromycin (ETM), Roxithromycin (ROM), Lincomycin (LM), Clindamycin (CLM), and Tylosin (TYL). The FQs included Danofloxacin (DAN), Sarafloxacin (SAN), Difloxacin (DIF), Enrofloxacin (ENR), Fleroxacin (FLN), Ciprofloxacin (CIP), Sparfloxacin (SPX), Norfloxacin (NOR), Pefloxacin (PFX), Ofloxacin (OFL), and Lomefloxacin (LMF). The TCs included Doxycycline (DC), Tetracycline (TC), Chlortetracycline (CTC), and Oxytetracycline (OTC). The SAs included Trimethoprim (TMP), Sulfamethoxazole (SMO), Sulfamethazine (SMH), Sulfadimethoxine (SMX), Sulfadiazine (SDZ), Sulfapyridine (SPD), Sulfathiazole (STZ), Sulfaquinoxaline (SQX), Sulfamonomethoxine (SMM), Sulfamethizole (SMT), Sulfachloropyridazine (SCP), and Sulfacetamide (SCT). The APs included Chloramphenicol (CHH), Thiamphenicol (THH), and Florfenicol (FF). The four internal standard compounds (ISTDs) included sulfadiazine-d6, levofloxacin-d8, tetracycline-d6, and chloramphenicol-d5. All the standards and ISTDs were purchased from Shanghai Aladdin Biochemical Technology Co., Ltd (Shanghai, China).

### 2.3. Sample Treatment

The sediment samples were freeze-dried under dark conditions and homogenized by passing through a 2 mm mesh sieve. The sediment samples (2 g) were weighed into a centrifuge tube, mixed with ISTDs (50 ng), and extracted with 20 mL of ACN/0.1 M EDTA-McIlvaine buffer (pH 4.0). The mixture was immediately vortexed for 2 min and ultrasonicated for 15 min. The solution was centrifuged at 6000 rpm for 5 min, and the resulting liquid above the sediment was gently moved to a clean centrifuge tube. The extraction process was repeated three times, and all the supernatants were combined and diluted to 250 mL with ultrapure water. Prior to solid-phase extraction, 0.4 g Na2EDTA was added to the extract to chelate the metal cations, and hydrochloric acid was added to adjust the pH of the water sample to 3.0. The cartridge was eluted using a solid-phase Oasis HLB (200 mg/6 cc) at a flow rate of 5 mL/min. The HLB cartridge was prepared by adding 5 mL of methanol and 5 mL of 0.1% formic acid. After loading the samples into the HLB cartridges, the column was purified with nitrogen for 20 min, eluted three times using 6 mL of methanol, dried under nitrogen, reconstituted in a 1.0 mL mixture of acetonitrile and water (1:1, *v*/*v*), and stored at −20 °C until HPLC analysis.

### 2.4. Instrumental Analysis

The HPLC-MS/MS utilized an Agilent 6410 B tandem triple-quadrupole LC-MS/MS with a Waters Xterra C18 separation column (100 mm × 2.1 mm, 3.5 μm) and an ESI ionization source. Mobile phase: phase A consisted of 0.1% formic acid and ammonium formate, whereas Phase B was composed of acetonitrile. Linear gradient: 0 min, 5% B; 0.1~10 min, 10~60% B; 10~12 min, 60%; and 12.1~22 min, 10% B. The flow rate was 0.25 mL/min. The temperature of the column was held at 25 °C, with an injection volume of 200 μL. The parameters for MS were set as follows: the temperature of the gas was 350 °C, with a flow rate of 8 mL/min; the nebulizer pressure was maintained at 25 psi; and the capillary voltage was set to 4000 V. 

### 2.5. Quality Control and Quality Assurance

The analyses underwent rigorous quality control measures. One parent ion and two sub-ion ions were selected for each compound for monitoring. Under the same experimental conditions, the absolute value of the relative standard deviation should be less than 3% when comparing the retention time of the compound to be tested with that of the target compound in the standard sample. Standard solution spectra with similar concentrations were used to compare the relative abundances of the qualitative ions of each compound. This difference did not exceed the relative standard deviation (0.81–2.74%). Before sample analysis, experimental blanks, procedure blanks, and blank spiked recoveries were established. 

### 2.6. Ecological Risk Assessment

The potential ecotoxicological risks of antibiotics in rivers were assessed using the risk quotient (RQ) [27,33]. The RQ values are typically represented as the ratio between the measured environmental concentrations (MECs) or predicted environmental concentrations (PECs) of pollutants and the predicted no-effect concentrations (PNECs) for those pollutants [34]. The RQ value is the measured environmental concentration (MEC) divided by the PNEC. 

The *PNEC_w_* was calculated according to the following equation (Equation (1)):

(1) $${PNEC}_{W}=\frac{NOEC\;or\;EC50}{AF}$$

where the no observed effect concentration and mean effective concentration are represented by *NOEC* and *EC*50, respectively. The assessment factor (*AF*) depends on toxicity data, with values of ten, fifty, or one hundred for chronic toxicity and one thousand for acute toxicity [34,35]. The most sensitive species were chosen to maximize the ecological impact of antibiotics. Without sediment toxicity data, the *PNEC* values were estimated from the *PNEC_w_* values using the equilibrium partition approach, as shown in the following equation (Equation (2)) [33]

(2) $${PNEC}_{S}={PNEC}_{W}\times K_{d}$$

where *K_d_* represents the sediment–water partition coefficient (L/kg) of antibiotics, as determined in previous studies [30,35]. The toxicity data for the antibiotics in this study were primarily obtained from previously documented sources, as detailed in Table 2.

The *RQ* values for evaluating the risks of antibiotics in the sediments were determined using the following equation (Equation (3)):

(3) $$RQ=\frac{{MEC}_{S}}{{PNEC}_{W}\times K_{d}}$$

where the *MECs* represent the concentrations of antibiotics in the sediments. Based on the *RQ* values, four levels of risk were defined: minimal risk (*RQ* < 0.01), low risk (0.01 ≤ *RQ* < 0.1), moderate risk (0.01 ≤ *RQ* < 1), and high risk (*RQ* ≥ 1) [27]. 

Antibiotics frequently occur in the natural environment in combinations, which can intensify their impact on the environment due to their combined effects [36]. Therefore, the combined risks should be calculated to evaluate the synergistic effects of antibiotics on biological systems [37]. A new combined *RQ* (*ΣRQ*) of antibiotics, utilizing the concentration coefficients of antibiotics as the weight assignments for evaluating the ecological risks of different antibiotics, was developed in a previous study [38]. Equation (4) is given as follows:

(4) $$\Sigma RQ=\Sigma \left({RQ}_{s}*{MEC}_{s}/{MEC}_{sum}\right)$$

where *ΣRQ* is the combined ecological risk of the 17 antibiotics, *RQs* is the ecological risk of each antibiotic, and *MEC_sum_* is the total concentration of antibiotics in the sediment samples (ng/g).

**Table 2:** Toxicity data of the antibiotics, including their *AF*, *PNEC_w_*, *K_d_*, and *PNEC_s_* values.

Antibiotics	Species	Toxicity Data (μg/L)	*AF*	*PNEC_w_* (ng/L)	*K_d_*	*PNEC_s_* (ng/g)	Ref.
AZM	*P. subcapitata*	*NOEC* = 10	50	200	17.3	3.46	[39,40]
ROM	*P. subcapitata*	*NOEC* = 10	10	1000	12	12	[27]
LM	*P. sucapitata*	*NOEC* = 5	10	500	5.4	2.7	[39,40]
CLM	*B. cifloru*	*EC_50_* = 24,940	1000	24,940	5.4	134.676	[39,40]
TYL	*D. polymorpha*	*NOEC* = 0.29	10	29	5.4	0.1566	[39,41]
ENR	*V. fischeri*	*NOEC* = 2.88	10	288	260	74.880	[27,42]
CIP	*L. perenne*	*NOEC* = 50	10	5000	417	2085.000	[39,43]
NOR	*M. aeruginosa*	*NOEC* = 1.6	10	160	537	85.92	[27,40]
OFL	*P. subcapitata*	*NOEC* = 1.13	10	113	1471	166.223	[39,44]
DC	*L. gibba*	*NOEC* = 10	10	1000	724	724	[39,40]
TC	*P. subcapitata*	*NOEC* = 0.5	10	50	1093	54.650	[27]
CTC	*L. gibba*	*NOEC* = 30	10	3000	778	2334.000	[39,40]
OTC	*E. densa*	*NOEC* = 20	10	2000	670	1340.000	[39,40]
TMP	*D. polymorpha*	*NOEC* = 0.29	10	29	7.4	0.2146	[39,45]
SMM	*C. vulgaris*	*EC_50_* = 5900	1000	5900	9.69	57.171	[27,29]
SCP	*L. gibba*	*EC_50_* = 2330	1000	2330	0.4	0.932	[39,40]
FF	*R. subcapitata*	*EC_50_* = 2300	1000	2300	29. 4	67.62	[27]

## 3. Results and Discussion

### 3.1. Occurrence and Composition of Antibiotics

The results from the antibiotic monitoring experiments carried out in the Anning River are displayed in Table 3 and Table S1. In general, the sediment samples detected 22 of the 36 antibiotics monitored. The recovery rate of antibiotics was 50.6~110.93%, the detection limit of the samples was 0.003~0.326 ng/L, and the quantitative line was 0.01~1.09 ng/L. At least four antibiotics were found at each sampling site, suggesting their widespread distribution in the Anning River. A total of 12 compounds, including LM, CLM, TYL, DAN, CIP, PFX, LMF, CTC, SMM, SMT, SCP, and SCT, were sporadically identified in the sediments at levels lower than 1 ng/g. As for the remaining ten antibiotics (AZM, ROM, ENR, NOR, OFL, DC, TC, OTC, TMP, and FF), their concentrations and detection frequencies (DFs) were relatively high.

According to Table 3, the total concentrations of antibiotics in surface sediments varied from 0.05 to 53.35 ng/g, with an average of 8.09 ng/g. The concentrations of SAs and APs ranged from <LOD to 39.54 ng/g and <LOD to 5.11 ng/g, respectively, with relatively low DFs of 40% and 70%, respectively. Contrastingly, MLs, FQs, and TCs exhibited the highest levels of DFs among the antibiotic categories. The concentrations ranged from <LOD to 6.39 ng/g (mean: 0.69 ng/g), 0.51 to 27.41 ng/g (mean: 2.1 ng/g), and <LOD to 7.98 ng/g (mean: 1.05 ng/g) for MLs, FQs, and TCs, with DFs of 80%, 100%, and 90%, respectively. In general, the sediment DFs were ordered as follows: FQs > TCs > MLs > APs > SAs. The variations in distribution were associated with the adsorption capacities, as compounds with greater adsorption coefficients were more commonly detected in the sediments. The distribution trend was consistent with the findings in Yangtze River sediments, showing a positive correlation between the distribution coefficients of these antibiotics and their *K_d_* values [27].

Additionally, FQs may also occur frequently due to their common usage in daily life. FQs are commonly utilized in human clinical medicine and animal breeding, whereas TCs are frequently utilized in veterinary medicine for prophylaxis and infection management, as well as for promoting animal growth due to their cost-effectiveness [7]. Among the FQs, OFL exhibited a concentration of 16.26 ng/g (mean: 4.18 ng/g), with a DF of 100%. ENR was typically found in concentrations ranging from <LOD to 1.44 ng/g (mean: 0.61 ng/g), with a DF of 90%. Meanwhile, the concentrations of four TCs ranged from <LOD to 4.76 ng/g, with DFs ranging from 20% (CTC) to 70% (doxycycline and tetracycline). In contrast, TMP from the SAs category exhibited the highest concentration at 39.49 ng/g (mean: 17.78 ng/g), with a DF of 30%.

The antibiotic concentrations observed in this study were generally lower than those reported in other river sediment studies. For instance, CIP, ENR, and NOR from the FQ category had concentrations of less than the limit of quantification (LOQ) of 44.23, 19.53, and 46.64 ng/g, respectively, in the Yangtze River [27]. Additionally, the concentration of TC in the Yangtze River (Chongqing section) ranged from <LOD to 5.03 ng/g, which was higher than the results obtained in this study. In contrast, the concentration of OTC in the Yangtze River was lower, ranging from <LOD to 2.59 ng/g [46].

When compared to other rivers in China (Table S2), the concentrations of TCs in this study were similar to those found in the urban rivers of Chengdu City, Sichuan Province (mean: 2.59 ng/g) [47]. However, the concentrations were notably lower than those in major rivers across China, such as the Pearl River (mean: 24.85 ng/g), the Yangtze River (mean: 25.95 ng/g), the Hai River (mean: 534.58 ng/g) [29], and the Hanjiang River (mean: 9.2 ng/g) [14]. Furthermore, the levels of MLs, FQs, and SAs in this study were found to be comparatively lower than those documented for other major rivers throughout China.

### 3.2. Spatial Distribution of Antibiotic Concentrations along the Anning River

**Figure 2:**
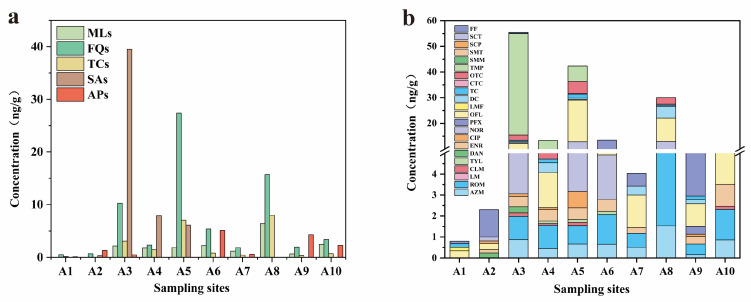
Concentration distribution of five antibiotic categories (**a**) and each antibiotic (**b**) in sediments from the Anning River.

The total concentrations of antibiotics in the sediment samples ranged from 0.80 to 55.51 ng/g (Figure 2a). There was no clear increasing trend in the sediment samples from upstream to downstream. The highest levels of antibiotic residues were found in midstream sediments, with average concentrations of 25.78 ng/g. This was followed by downstream concentrations of 15.36 ng/g and upstream concentrations of 1.55 ng/g.

The most polluted midstream area was found at site A3, where the total concentration in the sediments was 55.51 ng/g, dominated by TMP from the SAs category (Figure 2a,b). SAs are frequently employed in medical, agricultural, aquaculture, and livestock sectors to prevent and treat bacterial and protozoan infections [48]. Site A3 was located in the Anning Valley Great Plains, an area with developed animal husbandry and agriculture, suggesting a high likelihood of veterinary residue [49].

Another significantly polluted midstream area was observed at site A5, with total sediment concentrations reaching 42.43 ng/g. These concentrations were dominated by OFL and NOR in the FQ category. Both are used in human and veterinary pharmaceuticals [50], with a significant annual consumption of 5110 tons in China in 2013 [7]. Site A5 was located near Xichang City in the Anning Valley Great Plains, the largest city along the river. The potential sources of these antibiotics, due to dense population and economic growth, include wastewater treatment plants, sludge, and hospital wastewater associated with human activities [51,52,53].

However, no notable increase in concentrations was found in major cities along the river, such as Mianning (A2), Dechang (A7), and Miyi (A9). The most polluted downstream area was observed at site A8, with total concentrations in the sediments reaching 30.07 ng/g, dominated by OFL from the FQ category. OFL was the only compound detected at each sampling site. Its high absorption capability could be a significant contributor to its heightened detection levels [54]. OFL is primarily employed in the realm of human medicine, and its utilization is quite prevalent in China [16,55,56,57].

### 3.3. Ecological Risk Assessment

According to the risk assessment methods of the European Commission (2003), risk quotients (RQs) were estimated based on the predicted no-effect concentrations (PNECs) for the most sensitive species. The potential ecological risks of the antibiotics under investigation were evaluated based on toxicity data obtained from previous studies (Table 2). Out of the 22 compounds that were detected, ecological risk assessments could not be conducted for five (DAN, PFX, LMF, SMT, and SCT) due to insufficient toxicity data.

**Figure 3:**
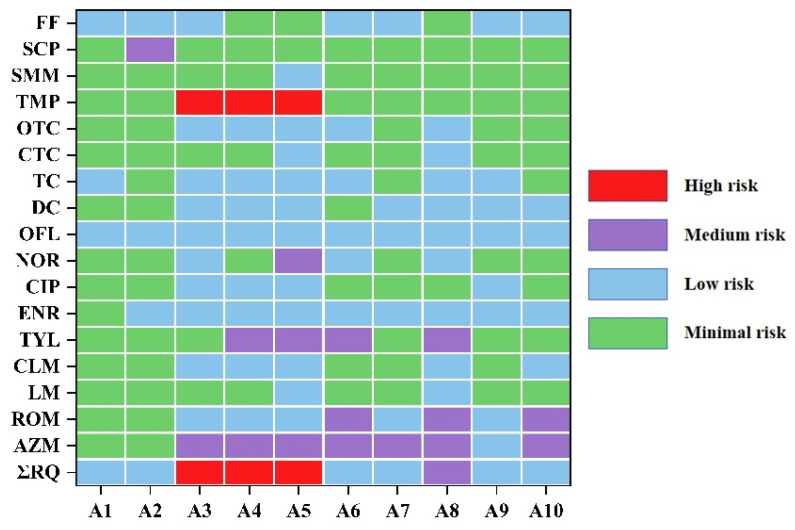
Ecological risks (RQs) of antibiotics in surface sediments from the Anning River.

The results are presented in Figure 3 and Table S3. Most antibiotics exhibited trace residues, resulting in RQs below 0.1, indicating low to minimal ecological risk. FQs, TCs, SAs, and APs generally pose minimal to low risks, suggesting that they may have a limited ecological impact on the Anning River. TMP from the SA group presented a high risk at sites A3, A4, and A5, with the highest concentrations at site A3. These findings indicate that TMP poses a high ecological risk to aquatic ecosystems and should be prioritized for control. OFL exhibited low risk at all the sampling sites, consistent with its 100% detection frequency.

The MLs, AZM, TYL, and ROM, were found to have medium risks at 70%, 40%, and 30% of the sampling sites, respectively. The high-risk sites were predominantly located in the midstream segment of the Anning River, in close proximity to residential and agricultural areas, whereas the medium-risk sites were found in both the midstream and downstream areas close to agricultural regions. The combined ecological risk (ΣRQ) of antibiotics was low at some sampling sites (sites A1–2, A6–7, and A9–10), with ΣRQ values ranging from 0.01 to 0.1 (Figure 3). The ΣRQ value at sampling site A8 indicated medium risk. High risks were noted at sites A3, A4, and A5, along the midstream area of the Anning River.

**Figure 4:**
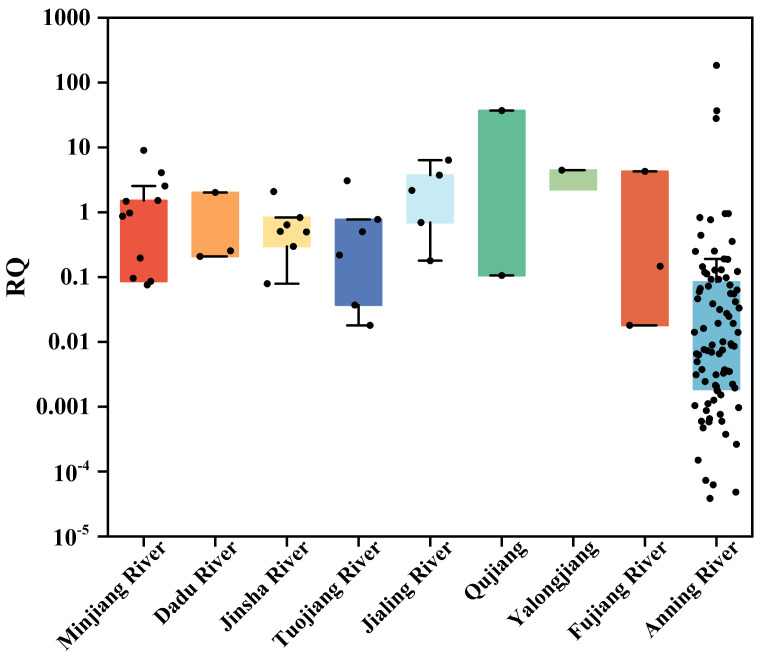
Ecological risks (RQs) of antibiotics from typical rivers in Sichuan Province [30] and the Anning River.

Upon comparing the ecological risk values of antibiotics in the typical rivers of Sichuan Province [30] with those in the Anning River, it was revealed that despite the highest value being present in the Anning River, the overall antibiotic risk values in this river were relatively low (Figure 4). However, the ecological risk level varies depending on factors such as the type of the antibiotic being tested, the quantity of sampling sites, and the duration of monitoring. Hence, further research is imperative to more accurately evaluate the ecological risk posed by antibiotics in the study area. 

Although the ecological risk associated with most antibiotics is considered to be low, there should be greater focus on the antibiotic risk to human health. Antibiotics in aquatic ecosystems can affect aquatic organisms, bacterial population dynamics, and the spread of antibiotic-resistant genes (ARGs) [58,59]. ARGs have the potential to be transmitted through horizontal gene transfer among different organisms, leading to the emergence of antibiotic-resistant bacteria that could pose a risk to human health through the food chain [60]. Unfortunately, there is limited data on the pollution characteristics and ecological risks of ARGs in the sediments of the Anning River. Further research in this area is necessary, and future ecological risk assessments of antibiotics in the environment should incorporate ARGs. Therefore, ecological risk assessment methods should continue to evolve and improve.

## 4. Conclusions

This study systematically investigated the occurrence, distribution, and ecological risks of 36 antibiotics in the surface sediments of the Anning River in Southwest China. A total of 22 antibiotics were detected in the sediments in the following order of abundance: FQs > TCs > MLs > APs > SAs. TMP from the SA group exhibited the highest concentration, reaching 39.49 ng/g (mean: 17.78 ng/g) at site A3, located in the Anning Valley Great Plains, where animal husbandry and agriculture are prevalent. The average concentrations of antibiotics in the Anning River were generally lower than those found in other studies of river sediments, including the Pearl, Yangtze, and Hai Rivers. The risk quotient method was used to assess the ecological risks of the detected antibiotics, and the results indicated that TMP from the SA group posed a high risk in the midstream section of the river (sites A3, A4, and A5), whereas AZM, TYL, and ROM from the ML group presented a medium risk. Compared to the RQ values observed in typical rivers within Sichuan Province [30], the ecological risk posed by antibiotics in the Anning River was relatively minimal. Nevertheless, further efforts are required to enhance the ongoing surveillance of antibiotic contamination within the study area. The findings of this study will contribute to bridging the existing knowledge gap regarding the antibiotic profiles in the surface sediments of the Anning River on a local scale. This information can contribute to future research on the long-term monitoring and risk control of the study area.

## Figures and Tables

**Table 1:** Basic information for each sampling site of the Anning River.

Sampling Site	Geographic Location (N, E)		County	Land Use	Site Characteristic
A1	28.66°	102.21°	Mianning	Forest	Upstream
A2	28.53°	102.18°		Built land	
A3	28.27°	102.18°		Cropland	Midstream
A4	27.95°	102.15°	Xichang	Cropland	
A5	27.84°	102.20°		Built land	
A6	27.51°	102.18°	Dechang	Cropland	
A7	27.36°	102.28°		Built land	
A8	27.10°	102.21°	Miyi	Cropland	Downstream
A9	26.81°	102.03°		Cropland	
A10	26.72°	101.85°		Forest	

**Table 3:** Concentrations (ng/g) of Antibiotics in Surface Sediments from the Anning River.

Antibiotics	Min	Max	Mean	DF ** (%)
AZM	<LOD *	1.53	0.71	80
ROM	<LOD	4.25	1.42	80
LM	<LOD	0.18	0.10	20
CLM	<LOD	0.30	0.17	50
TYL	<LOD	0.15	0.14	40
MLs	<LOD	6.39	0.69	80
DAN	<LOD	0.29	0.27	20
ENR	<LOD	1.44	0.61	90
CIP	<LOD	0.78	0.27	40
NOR	<LOD	9.64	4.90	40
PFX	<LOD	0.37	0.37	10
OFL	0.29	16.26	4.18	100
LMF	<LOD	0.19	0.18	30
FQs	0.51	27.41	2.10	100
DC	<LOD	4.56	1.08	70
TC	<LOD	1.5	0.45	70
CTC	<LOD	0.35	0.26	20
OTC	<LOD	4.76	2.16	50
TCs	<LOD	7.98	1.05	90
TMP	<LOD	39.49	17.78	30
SMM	<LOD	0.14	0.14	10
SMT	<LOD	0.05	0.05	10
SCP	<LOD	0.12	0.12	10
SCT	<LOD	0.19	0.19	10
SAs	<LOD	39.54	7.69	40
FF	<LOD	5.11	2.02	70
APs	<LOD	5.11	2.02	70

* <LOD: below the limit of detection. ** DF: detection frequency.

## Data Availability

All the data used in this work are available either within the article or in the Supporting Information File.

## Supplementary Materials

The following supporting information can be downloaded at: https://www.mdpi.com/article/10.3390/toxics12060411/s1, Table S1: Concentrations (ng/g) of antibiotics detected in the sediments of the Anning river; Table S2: The concentration of antibiotics in surface sediments in China (ng/g); Table S3: The risk quotients (RQs) of the target antibiotics in sediments from the Anning river. Reference [61] is cited in the Supplementary Materials.
