# Supplementary material for: Antibiotics in Surface Sediments from the Anning River in Sichuan Province, China: Occurrence, Distribution, and Risk Assessment
Source: Toxics. 2024 Jun 4;12(6):411. doi: 10.3390/toxics12060411 (PMC11209513; doi:10.3390/toxics12060411)
Supplement: Supplementary file 1 [file toxics-12-00411-s001.zip › toxics-3017406-supplementary.pdf]

Table S1 Concentrations (ng/g) of antibiotics detected in the sediments of the Anning river

Category	Compoud	Site									
		A1	A2	A3	A4	A5	A6	A7	A8	A9	A10
Macrolides (MLs)	Azithromycin(AZM)	<LOD	<LOD	0.87	0.45	0.66	0.65	0.5	1.53	0.16	0.86
	Roxithromycin(ROM)	<LOD	<LOD	1.11	1.11	0.87	1.42	0.67	4.25	0.5	1.46
	Lincomycin hydrochloride(LM)	<LOD	<LOD	<LOD	<LOD	0.01	<LOD	<LOD	0.18	<LOD	<LOD
	Clindamycin(CLM)	<LOD	<LOD	0.17	0.08	0.15	<LOD	<LOD	0.30	<LOD	0.14
	Tylosin (TYL)	<LOD	<LOD	<LOD	0.12	0.15	0.15	<LOD	0.13	<LOD	<LOD
Fluoroquinolones (FQs)	Danofloxacin(DAN)	<LOD	0.24	0.29	<LOD	<LOD	<LOD	<LOD	<LOD	<LOD	<LOD
	Enrofloxacin(ENR)	<LOD	0.16	0.49	0.56	0.55	0.57	0.28	1.44	0.37	1.05
	Ciprofloxacin (CIP)	<LOD	<LOD	0.13	0.08	0.78	<LOD	<LOD	<LOD	0.1	<LOD
	Norfloxacin(NOR)	<LOD	<LOD	2.72	<LOD	9.64	2.13	<LOD	5.12	<LOD	<LOD
	Pefloxacin(PFX)	<LOD	<LOD	<LOD	<LOD	<LOD	<LOD	<LOD	<LOD	0.37	<LOD
	Ofloxacin(OFL)	0.34	0.29	6.45	1.67	16.26	2.68	1.55	9.14	1.09	2.34
	Lomefloxacin(LMF)	0.17	<LOD	0.19	<LOD	0.18	<LOD	<LOD	<LOD	<LOD	<LOD

	Doxycycline(DC)	< LOD	< LOD	0.55	0.47	0.63	< LOD	0.43	4.56	0.19	0.7
Tetracyclines (TCs)	Tetracycline hydrochloride(TC)	0.17	< LOD	0.49	0.18	1.5	0.19	< LOD	0.47	0.17	< LOD
	Chlortetracycline hydrochloride(CTC)	< LOD	< LOD	< LOD	< LOD	0.17	< LOD	< LOD	0.35	< LOD	< LOD
	Oxytetracycline hydrochloride(OTC)	< LOD	< LOD	2.04	0.78	4.76	0.63	< LOD	2.6	< LOD	< LOD
	Trimethoprim(TMP)	< LOD	< LOD	39.49	7.88	5.98	< LOD	< LOD	< LOD	< LOD	< LOD
	Sulfamonomethoxine(SMM)	< LOD	< LOD	< LOD	< LOD	0.14	< LOD	< LOD	< LOD	< LOD	< LOD
Sulfonamides (SAs)	Sulfamethizole(SMT)	< LOD	< LOD	0.05	< LOD	< LOD	< LOD	< LOD	< LOD	< LOD	< LOD
	Sulfachloropyridazine(SCP)	< LOD	0.12	< LOD	< LOD	< LOD	< LOD	< LOD	< LOD	< LOD	< LOD
	Sulfacetamide(SCT)	< LOD	0.19	< LOD	< LOD	< LOD	< LOD	< LOD	< LOD	< LOD	< LOD
Amphenicols(APs)	Florfenicol(FF)	0.12	1.31	0.47	< LOD	< LOD	5.11	0.6	< LOD	4.27	2.25

Table S2. The concentration of antibiotics in surface sediments in China (ng/g).

Regions	Antibiotic Types and Concentrations in sediments (ng/g)				Ref.
	MLs	FQs	TCs	SAs	
Pearl River	n.d. ~ 385 (12.07)	n.d. ~ 1560 (139.82)	n.d. ~ 196 (24.85)	n.d. ~ 248 (3.23)	(Li et al., 2018)
Yangtze River	n.d. ~ 120 (12.63)	n.d. ~ 458 (8.31)	n.d. ~ 335 (25.95)	n.d. ~ 130 (9.43)	
Yellow River	n.d. ~ 49.8 (3.2)	n.d. ~ 142 (12.96)	n.d. ~ 184 (5.05)	n.d. ~ 22 (1.14)	
Hai River	n.d. ~ 5622 (55.43)	n.d. ~ 19591 (310.64)	n.d. ~ 162673 (534.58)	n.d. ~ 12300 (69.1)	
Liao River	n.d. ~ 229 (17.77)	n.d. ~ 362 (14.06)	n.d. ~ 653 (12.89)	n.d. ~ 9.84 (0.71)	
Urban river in Chengdu	n.d. ~ 6.3 (2.07)	n.d. ~ 62.9 (18.54)	n.d. ~ 10.8 (2.59)	n.d.	(Lyu et al., 2023)
Yongjiang River	0.09 ~ 2.58 (0.76)	/	/	n.d. ~ 0.81 (0.04)	(Xue et al., 2013)
main rivers of Chongqing city	n.d. ~ 866.78 (131.29)	n.d. ~ 15200 (830.83)	n.d. ~ 5.03 (2.22)	n.d. ~ 281.69 (22.05)	(Wang et al., 2020)
Hanjiang River	n.d.	n.d. ~ 9.9 (5.7)	n.d. ~ 14 (9.2)	n.d. ~ 10 (6.25)	(Hu et al., 2018)
lower Yangtze River	n.d. ~ 6780 (1200)	n.d. ~ 84170 (11622.5)	n.d. ~ 970 (430)	n.d. ~ 2040 (438)	(Zhang et al., 2020)
Aning River	n.d. ~ 4.25 (0.69)	n.d. ~ 16.26 (2.1)	n.d. ~ 4.76 (1.05)	n.d. ~ 39.49 (7.69)	This study

Table S3 The Risk quotient (RQs) of the target antibiotics in sediments from the Anning river

Compouds	Site									
	A1	A2	A3	A4	A5	A6	A7	A8	A9	A10
AZM	0	0	$2.514*10^{-1}$	$1.301*10^{-1}$	$1.908*10^{-1}$	$1.879*10^{-1}$	$1.445*10^{-1}$	$4.422*10^{-1}$	$4.624*10^{-2}$	$2.486*10^{-1}$
ROM	0	0	$9.250*10^{-2}$	$9.25*10^{-2}$	$7.25*10^{-2}$	$1.183*10^{-1}$	$5.583*10^{-2}$	$3.542*10^{-1}$	$4.167*10^{-2}$	$1.217*10^{-1}$
LM	0	0	0	0	$3.704*10^{-3}$	0	0	$6.667*10^{-2}$	0	0
CLM	0	0	$1.262*10^{-3}$	$5.94*10^{-4}$	$1.114*10^{-3}$	0	0	$2.228*10^{-3}$	0	$1.04*10^{-3}$
TYL	0	0	0	$7.663*10^{-1}$	$9.579*10^{-1}$	$9.579*10^{-1}$	0	$8.301*10^{-1}$	0	0
DAN										
ENR	0	$2.137*10^{-3}$	$6.544*10^{-3}$	$7.479*10^{-3}$	$7.345*10^{-3}$	$7.612*10^{-3}$	$3.739*10^{-3}$	$1.923*10^{-2}$	$4.941*10^{-3}$	$1.402*10^{-2}$
CIP	0	0	$1.000*10^{-4}$	$4.000*10^{-5}$	$3.741*10^{-4}$	0	0	0	$5.000*10^{-5}$	0
NOR	0	0	$3.166*10^{-2}$	0	$1.122*10^{-1}$	$2.479*10^{-2}$	0	$5.959*10^{-2}$	0	0
PFX						0				
OFL	$2.045*10^{-3}$	$1.745*10^{-3}$	$3.88*10^{-2}$	$1.005*10^{-2}$	$9.782*10^{-2}$	$1.612*10^{-2}$	$9.324*10^{-3}$	$5.499*10^{-2}$	$6.557*10^{-3}$	$1.408*10^{-2}$
LMF										
DC	0	0	$7.597*10^{-4}$	$6.491*10^{-4}$	$8.701*10^{-4}$	0	$5.939*10^{-4}$	$6.298*10^{-3}$	$2.624*10^{-4}$	$9.669*10^{-4}$
TC	$3.111*10^{-3}$	0	$8.966*10^{-3}$	$3.294*10^{-3}$	$2.745*10^{-2}$	$3.477*10^{-3}$	0	$8.6*10^{-3}$	$3.111*10^{-3}$	0
CTC	0	0	0	0	$1.000*10^{-4}$	0	0	$1.5*10^{-4}$	0	0
OTC	0	0	$1.522*10^{-3}$	$5.821*10^{-4}$	$3.552*10^{-3}$	$4.701*10^{-4}$	0	$1.94*10^{-3}$	0	0
TMP	0	0	$1.84*10^{2}$	3.672*10	2.787*10	0	0	0	0	0
SMM	0	0	0	0	$2.449*10^{-3}$	0	0	0	0	0

Compouds	Site									
	A1	A2	A3	A4	A5	A6	A7	A8	A9	A10
SMT										
SCP	0	$1.288*10^{-1}$	0	0	0	0	0	0	0	0
SCT										
FF	$1.775**10^{-3}$	$1.937*10^{-2}$	$6.951*10^{-3}$	0	0	$7.557*10^{-2}$	$8.873*10^{-3}$	0	$0.0631*10^{-2}$	$3.327*10^{-2}$

References

1. Yitao Lyu, Xuming Xu, Yibin Yuan, Zhaoli Wang, Jingrun Hu, Qian Chen, Weiling Sun, Antibiotic profiles and their relationships with multitrophic aquatic communities in an urban river, Science of The Total Environment, Volume 868, 2023, 161678,
2. Li S., Shi W.Z., Liu W., Li H.M., Zhang W., Hu J.R., Ke Y.C., Sun W.L., Ni J.R. A duodecennial national synthesis of antibiotics in China's major rivers and seas (2005–2016). Science of The Total Environment, 2018, 615: 906-917.
3. Wang, G.; Zhou, S.; Han, X.; Zhang, L.; Ding, S.; Li, Y.; Zhang, D.; Zarin, K. Occurrence, distribution, and source track of antibiotics and antibiotic resistance genes in the main rivers of Chongqing city, Southwest China. J. Hazard. Mater. 2020, 389, 122110.
4. Hu Y., Xue Y., Shen Y., Di M.X., Wang J. Antibiotics in surface water and sediments from Hanjiang River, Central China: Occurrence, behavior and risk assessmen. Ecotoxicology & Environmental Safety, 2018, 157: 150-158.
5. Guodong Zhang, Shaoyong Lu, Yongqiang Wang, Xiaohui Liu, Ying Liu, Jiamin Xu, Tingting Zhang, Zhi Wang, Yong Yang, Occurrence of antibiotics and antibiotic resistance genes and their correlations in lower Yangtze River, China, Environmental Pollution, 2020, 257, 113365.
6. Xue, B., Zhang, R., Wang, Y., Liu, X., Li, J., Zhang, G., 2013. Antibiotic contamination in a typical developing city in south China: occurrence and ecological risks in the Yongjiang River impacted by tributary discharge and anthropogenic activities. Ecotoxicol. Environ. Saf. 92, 229–236.
